# Two-Dimensional Material-Based Colorimetric Biosensors: A Review

**DOI:** 10.3390/bios11080259

**Published:** 2021-07-31

**Authors:** Danzhu Zhu, Bin Liu, Gang Wei

**Affiliations:** College of Chemistry and Chemical Engineering, Qingdao University, Qingdao 266071, China; zhudanzhu11@outlook.com (D.Z.); liubinname@outlook.com (B.L.)

**Keywords:** 2D materials, nanoparticles, nanozymes, hybrid materials, colorimetric biosensors

## Abstract

Two-dimensional (2D) materials such as graphene, graphene oxide, transition metal oxide, MXene and others have shown high potential for the design and fabrication of various sensors and biosensors due to their 2D layered structure and unique properties. Compared to traditional fluorescent, electrochemical, and electrical biosensors, colorimetric biosensors exhibit several advantages including naked-eye determination, low cost, quick response, and easy fabrication. In this review, we present recent advances in the design, fabrication, and applications of 2D material-based high-performance colorimetric biosensors. Potential colorimetric sensing mechanisms and optimal material selection as well as sensor fabrication are introduced in brief. In addition, colorimetric biosensors based on different 2D materials such as graphene, transition metal dichalcogenide/oxide, MXenes, metal–organic frameworks, and metal nanoplates for the sensitive detection of DNA, proteins, viruses, small molecules, metallic ions, and others are presented and discussed in detail. This work will be helpful for readers to understand the knowledge of 2D material modification, nanozymes, and the synthesis of hybrid materials; meanwhile, it could be valuable to promote the design, fabrication, and applications of 2D material-based sensors and biosensors in quick bioanalysis and disease diagnostics.

## 1. Introduction

The fabrication and application of high-performance sensors and biosensors have attracted great attention due to their high sensitivity and good selectivity [1,2,3], in which the colorimetric sensors exhibited a few advantages compared with other traditional electronic, electrochemical, fluorescent, and optical sensors, such as naked-eye determination and quick detection, without needing complicated instruments and with low cost [4,5,6]. Therefore, colorimetric sensors have been widely used for the quick detection of various analytes such as DNA, proteins, viruses, small molecules, metallic cations, and others [7].

In order to achieve the colorimetric functions and specific selectivity of the fabricated colorimetric sensors, functional nanomaterials or optical probes are usually needed [8,9]. Based on the type of materials used for the fabrication of colorimetric sensors, various mechanisms of colorimetric detection, such as surface plasmon resonance (SPR), nanozyme catalyzation, fluorescent on–off adjusting, ligand–receptor binding, and the color change of photonic crystals under stimulation, are now well known [7]. For instance, some noble metallic nanoparticles such as Au, Ag, and Cu have been utilized as good components for the fabrication of colorimetric sensors [10,11,12]. The addition of analytes into the nanoparticle system can induce the aggregation of nanoparticles and cause a color change due to the effect of SPR. With the development of nanotechnology and biotechnology, nanomaterial-based artificial biomimetic enzymes, named nanozymes, exhibited great potential for the fabrication of colorimetric sensors [13,14,15,16]. Nanozymes have similar enzymatic catalysis function to natural enzymes (such as oxidase and catalase) for promoting the reaction of H_2_O_2_ and 3,3′,5,5′-tetramethylbenzidine (TMB), resulting naked-eye-observed color change [17]. This technique can be used to fabricate various colorimetric sensors for small molecules and metallic ions as these analytes can inhibit the reaction by nanozymes and affect the color change of TMB.

Two-dimensional (2D) materials have been widely utilized for the fabrication of various colorimetric sensors and biosensors due to their special structure and properties, including their 2D layered structure, good conductivity, high mechanical stability, and facile functionalization [18,19,20]. First, the higher specific surface area properties of 2D materials can provide more active sites for binding analytes, which can improve the sensitivity of the fabricated colorimetric biosensors. Second, 2D materials show enhanced enzyme-mimic properties, and can be utilized for the fabrication of nanozyme-based colorimetric sensors and biosensors. Third, 2D materials can be used as excellent flat templates for conjugation with nanoparticles, quantum dots, polymers, biomolecules and others to form functional hybrid nanomaterials, which can improve the sensitivity and selectivity of the fabricated colorimetric sensors and biosensors. For instance, based on different inhibitory effects of ssDNA and dsDNA on the sedimentation behavior of WS_2_ nanosheets in sodium chloride solution, Ying et al. developed a colorimetric sensing strategy using 2D WS_2_ nanosheets for ssDNA detection [21]. In another study, Rostami et al. built a fast, simple, and sensitive graphene nanoribbon (GNR)/AgNP hybrid-based colorimetric sensor for the quantitative detection of dopamine (DA) and glutathione (GSH) based on the etching and aggregation process of AgNPs [22]. Othong et al. synthesized 2D metal–organic framework (MOF) nanosheets using the top-down liquid ultrasonic stripping technology, which exhibited the sensitive colorimetric determination of dimethylformamide in water [23].

In the review of Liu et al., the applications of 2D nanozyme materials in different biosensors are introduced, and the applications of colorimetric sensors, electrochemical sensors, fluorescent biosensors and electrochemiluminescence sensors are introduced, respectively [13]. Compared with colorimetric sensors, other biosensors have also been widely used. Electrochemiluminescence sensors use the transfer reaction of electrons to form a luminous emission state on the surface of the electrode, which has the advantages of low background emission and high sensitivity. Electrochemical sensors have the advantage of on-site detection, and they can realize the selective detection of different substances. In addition, fluorescent biosensors are based on the fluorescence emission of different materials, and a fast response to detection is realized by introducing the fluorescence signal into the detection system.

Previously, the fabrication and applications of colorimetric sensors and biosensors have been commented upon by some important reports. For instance, Priyadarshini et al. focused on the functionalization and applications of metal ion colorimetry, and further described the potential of nanoparticles as next-generation multifunctional sensing tools and as “lab on a chip” detection reagents [24]. The development of colorimetric detection also further promotes the development of colorimetric sensor arrays composed of colorimetric sensors. Liu et al. reviewed the development and status of AuNP-based colorimetric sensor arrays. In their review, the principles of sensor arrays are introduced briefly, and the design of mutual sensing element is discussed [25]. The microfabricated amperometric sensors and biosensors modified by photolithography can be amplified by photolithography, biomaterial or polymer, and the sensitivity of the electrode can be further improved. At present, microfabricated amperometric sensors and biosensors have been successfully applied to environmental testing, drug analysis, and biomedical applications [26]. In another case, Kangas and co-workers summarized how to carry out real-time identification of potential threats of weapons and explosives using colorimetric sensor arrays [27]. However, the design, fabrication, and applications of 2D material-based colorimetric biosensors have not been summarized, which we believe will be valuable for promoting the applications of 2D materials in analytical science, bioanalysis, and biomedicine.

In this review, we demonstrate recent research progress on the design, fabrication, and applications of 2D material-based colorimetric biosensors. For this aim, first we present the sensing mechanisms of various colorimetric sensors and then introduce potential materials and methods for colorimetric sensing. We specially focus on the design of colorimetric biosensors based on typical 2D materials, such as graphene, transition metal dichalcogenide/oxide (TMD/TMO), MXenes, MOFs, and 2D metal nanoplates. The cases on the detection of DNA, proteins, viruses, metallic ions, small molecules, and others by 2D material-based colorimetric biosensors are introduced and discussed in detail. Finally, the challenges and potential development of 2D materials for colorimetric analysis are presented.

## 2. Mechanisms and Materials of Colorimetric Sensing

Colorimetric sensing is highly related to the materials that are utilized for the fabrication of sensors. In this section, we would like to present various materials for the fabrication of colorimetric sensors and typical sensing mechanisms.

### 2.1. Colorimetric Sensors Based on SPR Change

Surface plasmon resonance (SPR) is the unique characteristic of metal nanostructures, and the SPR changes caused by the aggregation and decomposition of metal nanostructures can realize the manufacture of colorimetric sensors. Some metal nanoparticles such as AuNPs and AgNPs can show a highly strong color in the visible region, mainly due to local surface plasmon resonance (LSPR) [28]. LSPR comes from the collective oscillation of conduction band electrons caused by electromagnetic radiation interaction [29]. These nanoparticles [30] have a large molar extinction coefficient in the visible region, making them suitable probes for colorimetric analysis. Colorimetric sensing using SPR nanomaterials is usually based on the changes of different optical properties when metal nanoparticles are aggregated and dispersed.

Due to the characteristics of SPR, the monodispersed and aggregated nanoparticles reveal different colors. When the nanoparticles gather, the solution color of the nanoparticles will change, which is convenient for the naked eye to determine. This process of aggregation is caused by the composition of the solution or the properties of the surface of the particles, through the electrostatic interaction or hydrogen bonds between the nanoparticles and their surroundings [31].

**Figure 1:**
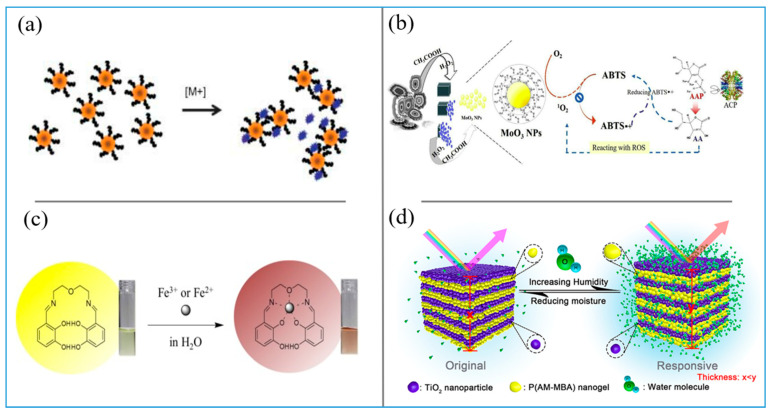
(**a**) Schematic illustration of the aggregation of peptide-functionalized nanoparticles (PFNs) in the presence of metal ions [M+]. Reprinted with permission from Ref. [33], Copyright 2008, Wiley-VCH. (**b**) Principle of ACP determination based on the oxidase-mimicking property of MoO_3_NPs. Reprinted with permission from Ref. [35], Copyright 2020, Elsevier. (**c**) Colorimetric Detection Mechanism of chelated Schiff base for Fe^2+^ and Fe^3+^. Reprinted with permission from Ref. [36], Copyright 2015, Elsevier. (**d**) Illustration of response of the 1DPC sensor to water vapor: the original 1DPC and the responsive 1DPC. Reprinted with permission from Ref. [37], Copyright 2018 American Chemical Society.

Based on the mechanism of SPR, the developed colorimetric sensors have been studied widely. For instance, Dash et al. used unmodified AgNPs to fabricate a colorimetric protein sensor [32]. Based on the unique SPR of AgNPs in dispersed and aggregated states, the colorimetric detection of proteins is realized. This method can sensitively detect proteins in the range of about 10.80 μg/mL. The Naik group found simple peptide ligands with rapid colorimetric reaction and detection limits within the nM concentration range (ppb) [33]. By using AuNPs that functionalized with peptides that can bind with metal ions specifically, the colorimetric detection of metal ions in solution was achieved. The binding of peptide ligands to metal ions leads to the aggregation of nanoparticles by bridging dispersed nanoparticles and by affecting the charge distribution of peptide coatings (Figure 1a). Zhou et al. developed a flow batch-operated AgNP-based automatic colorimetric sensor for the detection of trace Cu^2+^ in water samples [34]. Compared with previously reported colorimetric detection methods through the aggregation of AgNPs, this method had a lower detection limit, faster detection speed, and higher sensitivity to Cu^2+^. The linear range was 0.5–35 μg/L and the detection limit was 0.24 μg/L.

### 2.2. Colorimetric Sensors Based on Enzyme and Nanozyme Catalysis

According to the different types of enzymes, the common enzyme catalytic reactions can be divided into natural enzymes and mimic enzymes. There is no great difference between these two enzymes in catalytic mechanisms. Natural enzymes are usually accompanied with problems such as unstable catalytic activity and difficulties in preparation. However, the stability and catalytic activity of natural enzymes are excellent. Colorimetric sensors mediated by enzyme and mimic enzyme catalysis are usually based on color changes caused by enzyme and biomimetic enzyme catalysis of 3,3,5,5-tetramethylbenzidine (TMB) [7].

Taking the simplest hydrogen peroxide (H_2_O_2_) as an example, as H_2_O_2_ is involved in the oxidation of TMB in the catalase-like reaction, the concentration of H_2_O_2_ can be quantified by colorimetry [38]. For the colorimetric detection mechanism of glucose, glucose can be oxidized in the presence of glucose oxidase (Gox) to produce H_2_O_2_ products [39,40]. Therefore, a peroxidase mimic catalytic reaction has been used to indirectly detect glucose. For the typical detection of small molecules, such as dopamine, amino acid, etc., it is well known that the reaction of peroxidase and oxidase is the process of substrate flower cultivation, which makes use of the ability of reducing small molecules to inhibit the oxidation process, which has been utilized to achieve the colorimetric sensing of small molecules.

Li et al. proposed a one-pot non-enzyme strategy for the colorimetric detection of glucose [41]. The 2D metal oxides as nanozymes were synthesized under the guidance of bovine serum albumin, in which the oxidation of glucose and the colorimetric detection of H_2_O_2_ were carried out together under the catalysis of a single nanoenzyme. This detection method can not only realize the rapid qualitative analysis of glucose by the naked eye, but also quantitatively analyze glucose by spectrophotometry. Chen and co-workers found that MoO_3_NPs have the property of oxygen scanning because they can promote the rapid oxidation of 2,20-azino-bis (3-ethylbenzothiazoline-6-sulfonicacid (ABTS), 3,30,5,50-tetramethylbenzidine (TMB) and o-phenylenediamine (OPD) without the participation of H_2_O_2_, by which they designed a colorimetric sensor for the detection of acid phosphatase (ACP) [35]. The detection mechanism is based on (AA). The products of the ACP–ascorbic acid phosphate (AAP) system can interfere with the oxidation of ABTS and inhibit the activity of MoO_3_NP oxidase, as shown in Figure 1b.

### 2.3. Colorimetric Sensors Based on Fluorescence Switch

The principle of the colorimetric sensor via fluorescence switch is usually based on the fluorescence properties of some organic dyes, fluorescent polymers, and fluorescent nanomaterials (such as metal nanoclusters, quantum dots, and carbon dots). As a result, some colorimetric sensors with analyte-induced fluorescence switching behavior are fabricated [7]. Based on the behavior of fluorescence quenching switch, colorimetric detection can usually be realized using the following behaviors. For instance, by adding the analyte of quenching dye fluorescence, the fluorescence chromophore can be changed from “on” to “off” [42]. Secondly, the color change from “off” to “on” can be realized by the induced emission of a fluorescent polymer [43]. In addition, the “on–off–on” mode can be realized by designing a specific fluorescent probe, in which the addition of the first chemical substance (analyte) reacts with the fluorescent probe, resulting in an “on–off” change. After that, another analyte is added to restore the fluorescence of the probe through the “off–on” mode [44].

Yang et al. designed and synthesized chromone Schiff base derivatives as a fluorescent colorimetric probe to recognize Al^3+^ in ethanol [45]. Compared with other competitive ions in ethanol, the Schiff base probe showed a significant “on” fluorescence response to Al^3+^. According to this sensing mechanism, the detection of Al^3+^ on filter paper was achieved successfully. Liu et al. designed and fabricated a new fluorescence and colorimetric platform for the simple and selective detection of iodine ions in gold/silver nanoclusters (DNA-Au/AgNCs) on DNA templates [46]. The detection mechanism is based on the interaction between iodine ions and DNA-Au/AgNCs. The addition of iodine ions leads to fluorescence quenching, and the detection system of the solution changes from colorless and transparent to purplish red, thus realizing the colorimetric detection of iodine ions. In addition, the design of the fluorescent “off–on–off” mechanism can realize the specific recognition of different ions. For example, Wang et al. designed a colorimetric sensor for the detection of fluoride and copper ions using anion–cationic relay recognition [47]. The detection mechanism is based on the fluorescence “off–on–off” mechanism to achieve unprecedented sequence-specific sensing of fluoride and copper (II) ions.

### 2.4. Colorimetric Sensors Based on Ligand–Receptor Binding

The interaction between ligands and receptors as indicators (fluorophore and chromophore) and analytes (cation/anion) can change the emission spectrum of the indicator and provide a colorimetric response to different concentrations of analyte. The most common ligand or receptor is a Schiff base, which has been widely used in the manufacture of colorimetric chemical sensors to detect metal cations and other anions.

Among many colorimetric sensors based on receptor and ligand binding, Schiff bases are widely used in the research of colorimetric sensors. Schiff bases are good ligands for metal ions—especially chelated Schiff base derivatives that are obtained from the combination of 2-dihydroxybenzaldehyde and oxygen-bridged diamine have been proved to be promising tools for the optical sensing of metal ions. A series of colorimetric sensors based on Schiff bases has been developed for the detection of metal ions. You et al. synthesized a chelating chemical sensor based on the combination of oxygen-bridged diamine and two dihydroxyphenyl groups for the colorimetric detection of different metal ions [36]. The chelated Schiff base changed from yellow to brown in aqueous solution and showed high selectivity and sensitivity to Fe^2+^ and Fe^3+^ colorimetric recognition (Figure 1c). In other cases, multifunctional Schiff bases have been utilized for the colorimetric detection of Cu^2+^ [48], Al^3+^, and CN^−^ [49].

### 2.5. Colorimetric Sensors Based on Photonic Crystals (PCs)

PC is a kind of material with a periodic refractive index change, which has the characteristic of a photonic band gap (PBG), which can make some wavelengths in the PBG difficult to pass through. Therefore, PCs have been used in optical fibers, displays, sensors and other fields [50,51]. PCs can also be used in the field of colorimetric sensing due to their unique structure and color change. The detection principle of PC-based colorimetric sensors is to produce discoloration materials that have influence on the change in environment based on the color change of the PC structure. The colorimetric sensor can transform the change in the environment into the change in visual color, so as to develop a sensor with low cost and low power consumption [50,52]. The existence of a PBG can provide an intuitive, simple but powerful monitoring mechanism [53].

Paterno et al. reported a high-performance colorimetric sensor of bacterial contaminants by 1D PCs [54]. The color purity of PCs allows this offset to be translated into visible light in the UV region, which makes it easy to read pollution by colorimetry. This test is more sensitive to Gram-negative bacteria than Gram-positive bacteria, thanks to the fact that the outer membrane of Gram-negative bacteria is negatively charged, which makes it easier to interact with positively charged silver ions and adhere to cells. Tang et al. reported the synthesis of a mesoporous and colored PC by assembling polyacrylamide-methylene bis (acrylamide) (P(AM-MBA) nanogel and titanium dioxide (TiO_2_) nanoparticles together, which could be used as a high-performance colorimetric humidity sensor [37]. The principle and structure of the PC-based colorimetric sensor and its sensing mechanism are shown in Figure 1d. In this design, water-sensitive nanogels and mesopores were combined into the PC structure to achieve a fast, easy to identify, stable, and reversible humidity response.

### 2.6. Summary

In order to assist the understanding of the above introduction and discussion, we summarize the colorimetric detection mechanism of various colorimetric sensors and the used materials, as well as their specific detection applications, as shown in Table 1. The contents are classified and summarized from five aspects: SPR changes, enzyme and mimic enzyme catalysis, fluorescence switches, ligand and receptor binding, and PCs.

## 3. Fabrication and Applications of 2D Material-Based Colorimetric Biosensors

In this section, we present the design, fabrication, and applications of colorimetric biosensors using 2D materials, including graphene, TMD/TMO, MXenes, MOFs, and metal plates as functional building blocks. The highly sensitive detection of various analytes and corresponding sensing mechanisms are introduced and discussed.

### 3.1. Graphene-Based Colorimetric Biosensors

Graphene and graphene oxide (GO) have a unique ability to adsorb biomolecules such as nucleic acids, proteins, and peptides. In addition, they have strong fluorescence quenching and the ability to distinguish ssDNA and dsDNA. Graphene is a good substrate material for the design and fabrication of colorimetric sensors and biosensors. The simple, fast and efficient colorimetric detection method combined with the excellent ability of graphene can design faster and more sensitive colorimetric sensing platforms for the detection of specific biomolecules.

#### 3.1.1. Detection of Protein

**Figure 2:**
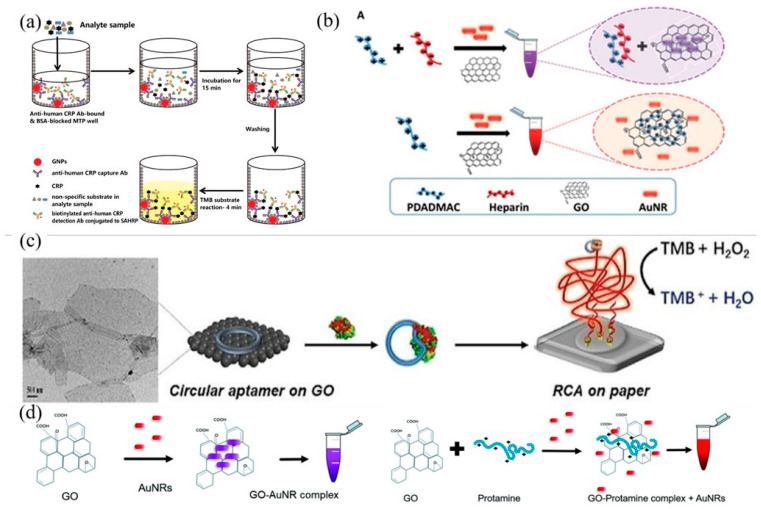
(**a**) GNP/MTP detection CPR. Reprinted with permission from Ref. [55], Copyright 2013, Elsevier. (**b**) PDADMAC is probe colorimetric detection of heparin. Reprinted with permission from Ref. [56], Copyright 2019, Royal Society of Chemistry. (**c**) Circular aptamer-modified graphene colorimetric detection of protein. Reprinted with permission from Ref. [57], Copyright 2019, Royal Society of Chemistry. (**d**) GO/AuNR hybrids for colorimetric detection of protamine. Reprinted with permission from Ref. [58], Copyright 2019, Royal Society of Chemistry.

Graphene can be functionalized based on the adsorption capacity of graphene to protein and nucleic acids to obtain a higher specificity of graphene-based materials. By means of KOH pretreatment, Vashist and colleagues covalently linked a graphene nano-platelet (GNP) to a polystyrene microtitration plate (MTP) using silane coupling agent APTES to synthesize a colorimetric sensing platform rich in human C-reactive protein (CRP) antibodies [55]. As shown in Figure 2a, the analytical sample is dripped onto GNP/MTP, and after incubation at 37 °C for 15 min, CPR binds with antibodies to make GNP/MTP have catalytic oxidation activity, resulting in a significant color change in TMB.

Based on the behavior that the aggregation of gold nanorods (AuNRs) on the surface of GO will lead to the change in solution color, Bamrungsap et al. proposed a colorimetric strategy for the detection of heparin [56]. Using poly (diallyl dimethyl ammonium chloride) (PDADMAC) as a probe (Figure 2b), the combination of PDADMAC with graphene prevents the aggregation of AuNRs on the surface of graphene, and the solution is red. However, when heparin appears, heparin binds with PDADMAC to form a stable polymer, and AuNRs are self-assembled on the surface of graphene to make the solution purple, thus achieving the purpose of colorimetric detection of heparin.

Li et al. found that circular aptamers can bind to graphene through π–π interaction [57]. When they encounter targeted protein targets, the configuration of circular aptamers bound to graphene will change, resulting in isothermal amplification. As shown in Figure 2c, the ring aptamer bound with graphene produces a rolling ring amplification reaction on the paper surface, amplifies the peroxidase activity, and has the function of the catalytic oxidation of TMB, so that the solution produces a significant color change so as to achieve the purpose of protein detection.

Using the binding of graphene and metal ions, Wiriyachaiporn and colleagues synthesized GO/AuNR hybrids for the colorimetric detection of protamine by the self-assembly of AuNRs on the surface of GO. As shown in Figure 2d, when AuNRs combine with GO nanosheets through electrostatic interaction, the color of the solution will change from red to dark purple due to the aggregation of AuNRs. When protamine is added, it will inhibit the aggregation of AuNRs, so that AuNRs remain dispersed, and the solution turns red again to achieve the detection purpose [58].

#### 3.1.2. Detection of Metal and Non-Metallic Ions

Metal ions can be colorimetrically detected by 2D material-based sensing platforms. Liu et al. used polyaniline (PEI) as the connector between metal ions and graphene to disperse Pd NPs on the surface of graphene [59]. In the presence of Hg^2+^, the peroxidase mimetic activity of the formed 2D rGO/PEI/Pd nanohybrids was enhanced greatly, which promoted the oxidation and color change of TMB to dark blue. Compared with the traditional Pd NPs for Hg^2+^ detection, the activity of 2D rGO/PEI/Pd nanohybrids is higher and the detection limit is lower. Borthakur and colleagues prepared a cobalt sulfide (CoS)-modified GO nanocomposite by the solvothermal method for the detection of Hg^2+^ in water [60].

**Figure 3:**
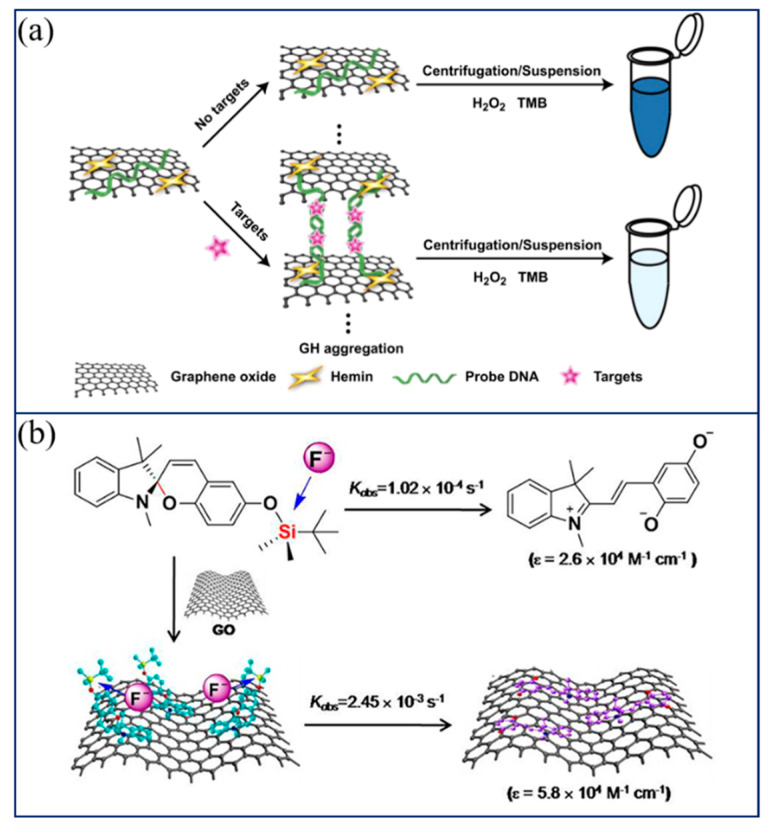
(**a**) Schematic illustration of procedures for Hg^2+^ detection. Reprinted with permission from Ref. [61], Copyright 2013, Elsevier. (**b**) Determination of fluoride by functionalized graphene. Reprinted with permission from Ref. [62], Copyright 2013, American Chemical Society.

It has been reported that some analytes, including metallic ions, DNA, and other small molecules, can be detected by combining hemin-modified GO with a DNA probe [61]. As shown in Figure 3a, hemin is bound to graphene by the DNA-mediated synthesis method, and different optical signals are generated according to the concentration of targets, so as to achieve a simple and fast colorimetric detection of various analytes.

In addition to metal ions, functionalized graphene can also be used for the colorimetric detection of non-metallic ions. For instance, Li et al. designed a rapid and sensitive sensor for the detection of fluoride ions in water by self-assembling fluoride ion-specific silyl-appended spiropyran dye on GO [62]. As shown in Figure 3b, the nucleophilic substitution reaction between the dye molecules and fluoride ions leads to the cleavage of the Si–O bond, resulting in the opening of the closed spiropyran structure and the color change from a colorless to an orange-yellow solution. With the addition of GO, the range of change is shortened, the detection limit for fluoride ions is lower, and the sensitivity is also improved.

#### 3.1.3. Detection of Viruses and Bacteria

Previously, functional nanomaterial-based sensing platforms have been widely used for the colorimetric detection of pathogenic bacteria and viruses [5]. Graphene and graphene-based hybrid materials have shown a wide range of applications in virus detection. Zhan et al. used a mercury-catalyzed AuNP–GO hybrid system to realize the colorimetric detection of respiratory virus (RSV) [63]. In their study, tannic acid (TA) was used to disperse GO nanosheets in solution, and then chloroauric acid (HAuCl_4_) was added to form AuNPs on the GO surface. The formed AuNP–GO nanohybrids exhibited peroxidase-like activity, which could be decreased after adding the corresponding antibodies. After the combination of RSV, the peroxidase-like activity of AuNP–GO was restored by adding Hg^2+^ to the solution. Based on this sensing mechanism, AuNP–GO-based colorimetric detection of RSV that was catalyzed by Hg^2+^ was achieved.

**Figure 4:**
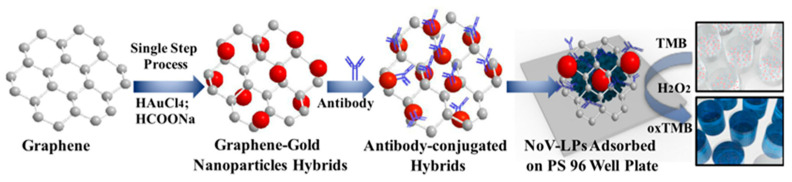
Antibody-modified graphene/AuNP hybrids for detection of norovirus (NOV-LP). Reprinted with permission from Ref. [64], Copyright 2017, Elsevier.

Ahmed and colleagues demonstrated the synthesis of antibody-modified graphene/AuNP hybrids for the colorimetric detection of norovirus (NOV-LP) [64]. As shown in Figure 4, the attachment of AuNPs onto the graphene surface facilitates the binding of antibodies to the formed graphene/AuNP hybrid materials, forming a nanoprobe after binding with virus antibodies. In the TMB/H_2_O_2_ system, the addition of NOV-LP changed the color of TMB solution to blue, which is sensitive and rapid to achieve the purpose of the detection of the virus. In another case, Guo et al. synthesized hemin-modified graphene nanosheets by a wet chemical method and π–π interaction [65]. The formed graphene-based hybrid materials could be utilized as a simple, convenient and environmentally friendly colorimetric platform for label-free detection of Hepatitis B virus (HBV).

Based on the specific binding between bacteria and antibodies or ssDNA aptamers, the colorimetric detection of bacteria can also be carried out on 2D material-based sensing platforms. For instance, Kaushal et al. reported the synthesis of a GO and AuNP-based colorimetric sensor for the rapid detection of *E. coli* and *S. typhimurium* by the assistance of antibodies [66]. In another study, Wu and co-workers reported the synthesis of ZnFe_2_O_4_–rGO hybrid nanostructures, which could serve as an effective peroxidase nanozyme for the colorimetric detection of *S. typhimurium* [67]. For this aim, the aptamer was bound onto the ZnFe_2_O_4_–rGO hybrid and acted as a single probe, and another biotin-aptamer was used as the capture probe. After adding the bacteria target, a sandwich structure was formed. Due to the high peroxidase-like activity of the ZnFe_2_O_4_–rGO nanozyme towards the catalysis of TMB, the TMB/H_2_O_2_ system revealed a clear color change to dark blue. The fabricated colorimetric sensor exhibited a detection limit of 11 CFU/mL with a detection range from 11–1.10 × 10^5^ CFU/mL for *S. typhimurium* in buffer solution.

**Figure 5:**
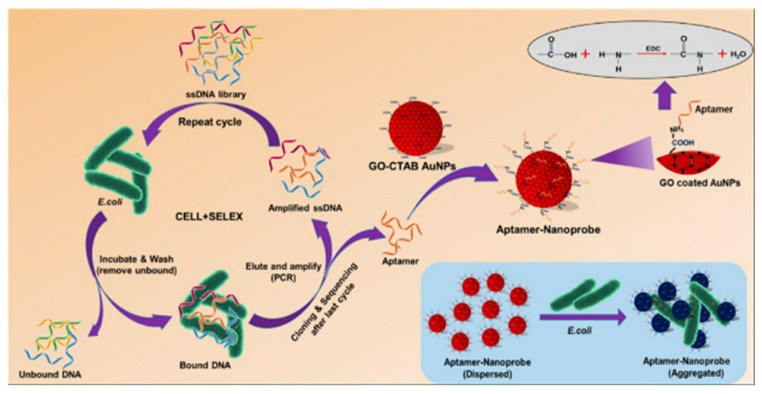
Colorimetric detection of Escherichia coli with GO-coated AuNPs and binding of aptamer. Reprinted with permission from Ref. [18], Copyright 2021, Elsevier.

Very recently, Gupta and colleagues designed an effective and flexible colorimetric method for the colorimetric detection of *E. coli* using ssDNA aptamers to modify GO that was wrapped in AuNPs, which is of great significance for the control of some diseases [18]. As shown in Figure 5, GO is coated on the surface of AuNPs to enhance the binding of graphene to aptamers, and E8 aptamers with high affinity are selected to bind suitable aptamers onto the surface of graphene through the coupling of AuNPs. In the presence of bacteria, the aptamer binds to the target bacteria on the cell surface and absorbs AuNPs, which leads to the aggregation of AuNPs and color change. The obvious change from red to blue in the solution can be observed directly by the naked eye. In the absence of target bacteria, AuNPs remain dispersed and the solution remains its original red.

#### 3.1.4. Detection of DNA/RNA

**Figure 6:**
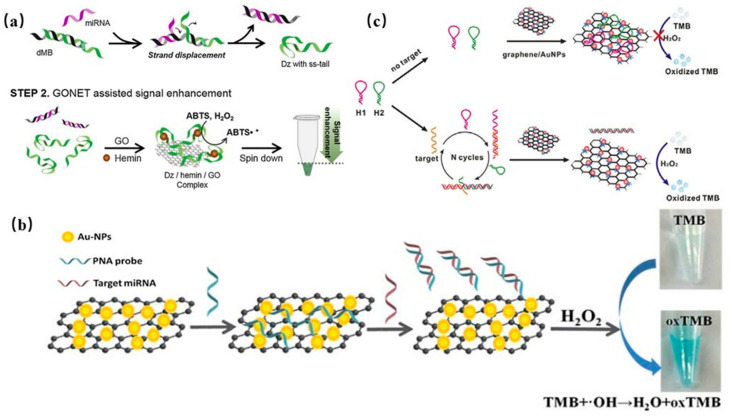
Colorimetric sensing DNA and RNA with 2D materials: (**a**) Colorimetric detection of graphene by miRNA network. Reprinted with permission from Ref. [68], Copyright 2019, Elsevier. (**b**) Graphene/AuNP hybrid material detection by miRNA. Reprinted with permission from Ref. [69], Copyright 2016, Royal Society of Chemistry. (**c**) Graphene/gold hybrid material detection by DNA. Reprinted with permission from Ref. [70], Copyright 2016, Elsevier.

MiRNA is a genetic marker of many diseases. The rapid and efficient detection of miRNA is very effective for the detection and control of some diseases. The traditional miRNA detection method is very complex and expensive. Lee and colleagues developed a network strategy for the graphene-based colorimetric detection of miRNA with high sensitivity [68]. Through graphene network capture and collection of miRNA, an miRNA/graphene composite network was built, which improved the colorimetric performance. Using a double molecular information marker to chain exchange with miRNA, the color change in colorimetric detection was improved, as shown in Figure 6a.

Zhao et al. developed a label-free colorimetric sensor for miRNA detection based on the spontaneous adsorption of PNA-21 on a graphene/AuNP hybrid surface. As shown in Figure 6b, the accumulation between the PNA probe and graphene completely inactivates the peroxidase-like catalysis of the graphene/AuNP, which greatly decreases the catalytic oxidation of TMB by H_2_O_2_. Therefore, the addition of miRNA triggered the hybridization reaction between ssPNA-21 and miRNA, which caused PNA to be released from graphene/AuNP, restoring the catalytic activity of graphene/AuNP, accompanied by a colorless to blue color change [69].

Chen and co-workers proposed a strategy to assemble a graphene/gold hybrid platform of (CHA) based on targeted catalytic hairpin assembly to achieve the label-free detection of DNA. As shown in Figure 6c, H1 and H2 cannot be assembled without a target. Graphene/gold hybrid material adsorbs H1 and H2, and its catalytic activity was inhibited. Therefore, the oxidization of TMB was inhibited. With the addition of targeted DNA, the hairpin assembly started and the H1–H2 assembly began, which decreased the binding affinity with graphene/gold hybrid materials, producing a colorless to blue color change [70].

#### 3.1.5. Detection of Small Molecules

Two-dimensional material-based colorimetric biosensors can also be utilized for the high-performance detection of small molecules, such as small molecule drugs, small biomolecules, organic molecules, and others.

For instance, Yuan et al. developed a colorimetric method for the determination of a small molecule drug, oxytetracycline, based on the controllable peroxidase activity of graphene/gold hybrid materials and the oxytetracycline aptamer [71]. As the graphene/gold hybrid has the ability to adsorb ssDNA through π–π interaction, the combination of the two components can block the peroxidase-like activity of the graphene/gold hybrid material. When oxytetracycline is added, the aptamer–oxytetracycline complex automatically forms and escapes from the hybrid, and the graphene/gold hybrid material recovers the peroxidase-like activity, resulting in color change and achieving the detection purpose.

Wang and co-workers used graphene as a transparent and efficient carrier of a polydiacetylene (PDA)-based colorimetric sensor, which exhibited high performance for sensing volatile organic compounds (VOCs) via observing the color change of the solution from blue to red [72]. This method has high sensitivity, a short response time, and a strong colorimetric signal, which can be used to detect low concentration VOCs.

**Figure 7:**
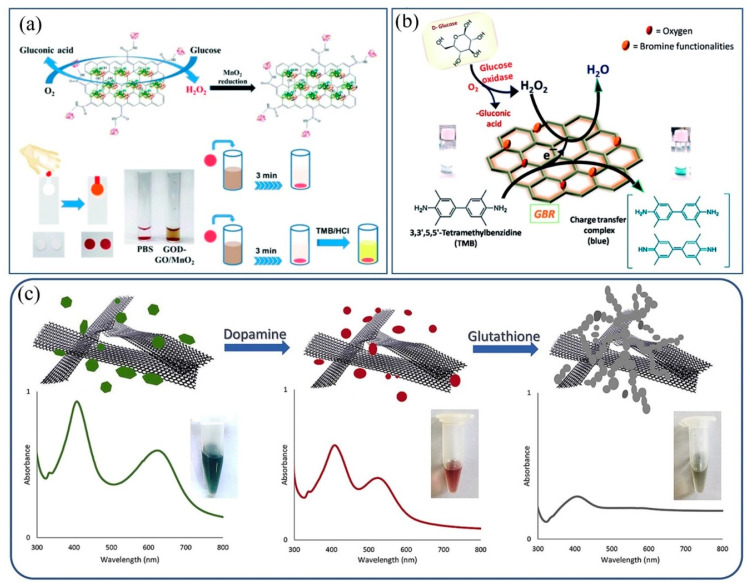
Colorimetric sensing of small molecules with 2D materials: (**a**) oxidase-graphene oxide/manganese dioxide (GOD-GO/MnO_2_) direct colorimetric determination of glucose concentration. Reprinted with permission from Ref. [74], Copyright 2019, Royal Society of Chemistry. (**b**) Plausible mechanism of glucose detection. Reprinted with permission from Ref. [75], Copyright 2017, Royal Society of Chemistry. (**c**) Graphene/silver hybrid material colorimetric detection of dopamine (DA) and glutathione (GSH). Reprinted with permission from Ref. [22], Copyright 2020, Elsevier.

Sharma et al. hybridized CuO with graphene to obtain CuO–GO nanospheres for the colorimetric detection of H_2_O_2_, which can also be used to detect cholesterol because of the production of H_2_O_2_ during the oxidative decomposition of cholesterol [73]. Lee et al. reported the synthesis of GO/MnO_2_ hybrid material by embedding MnO_2_ NPs onto bovine serum albumin (BSA)-coated GO, which could act as a novel nanozyme with oxidase activity [74]. As presented in Figure 7a, a dual sensing platform based on the combination of plasma separation and GOD-GO/MnO_2_ was constructed for direct colorimetric determination of glucose concentration without the pretreatment of the blood sample. Singh and colleagues developed a kind of brominated graphene for the colorimetric detection of glucose and H_2_O_2_ [75]. As shown in Figure 7b, the created brominated graphene revealed that peroxidase-like activity can catalyze the decomposition of H_2_O_2_ and oxidize TMB to make the solution blue.

Lin proposed an in situ method for the synthesis of PtNPs on GO to synthesize PtNPs/GO hybrids for the fabrication of a colorimetric cysteine sensor [76]. The catalytic oxidation activity of PtNPs/GO hybrid materials is significantly higher than that of other Pt-based peroxidase mimics. The adsorption of PtNP oxidation products by cysteine can block the active site of PtNPs and inhibit the peroxidase-like activity of PtNPs/GO. Therefore, the oxidation of TMB was hindered and the solution color was changed, so as to achieve the detection of cysteine. Rostami and colleagues hybridized graphene nanosheets with AgNPs by a plasma-mediated hybridization technique and established a graphene/AgNP-based colorimetric sensor, which exhibited high performance for the detection of dopamine (DA) and glutathione (GSH) in human serum samples (Figure 7c) [22]. The morphology of graphene/AgNP hybrids changes during etching, and the solution color changes from green to red, thus realizing the detection of DA. Subsequently, GSH induced the aggregation of AgNPs, resulting in a decrease in the absorption intensity of the SPR band of AgNPs. The color of the solution changed from red to gray, so that GSH can also be effectively detected by naked-eye observation.

Based on the above introduction and discussion, it can be found that GO containing other biosensing probes (such as metal nanoparticles, enzymes, and aptamers) is more suitable as a platform for colorimetric biosensing. GO has only very limited nanozyme catalytic activity for the fabrication of TMB/H_2_O_2_-based colorimetric biosensors or SPR-based biosensors. To overcome this problem, it is possible to synthesize hybrid GO-based composite materials through conjugation with other nanoscale biosensing probes, such as metal nanoparticles, nanorods, and metal oxide nanoparticles. It is clear that the addition of biosensing probes improved the sensing performance (detection limit, sensitivity, and selectivity).

### 3.2. TMD/TMO

Two-dimensional TMD materials such as tungsten disulfide (WS_2_), molybdenum disulfide (MoS_2_), tungsten diselenide (WSe_2_), and molybdenum diselenide (MoSe_2_) have excellent optical and catalytic properties. The optical induction properties of metal ions in TMD nanowires, such as WS_2_, MoS_2_, WSe_2_ and MoSe_2_, increase in plane size and thickness, which leads to the change in the characteristic absorption peak. Based on the different affinity of TMD to ssDNA and dsDNA, as well as the different performance in inhibiting the deposition of TMD nanowires in NaCl solution, Lan and co-workers reported a sensitive, label-free ssDNA colorimetric detection platform [21]. The sensing mechanism is similar to the colorimetric sensing mechanism induced by the SPR change of metal nanoparticles. To improve the sensitivity, polymerized chain reaction was introduced to achieve a higher performance in DNA detection and a wider detection range.

As a 2D TMD material with peroxidase-like activity, MoS_2_ can be hybridized with inorganic metal nanomaterials to improve the nanozymatic activity and extend the application prospect. For instance, Wan and colleagues studied the catalytic oxidation performance of MoS_2_-Au@Pt nanosheets that were synthesized by conjugating MoS2 nanosheets with bimetallic Au@Pt NPs [77]. It was found that cysteine can effectively block the oxidation of TMB and inhibit the catalytic activity of MoS_2_-Au@Pt nanozymes, resulting in the color change of the solution from dark blue to colorless in the presence of cysteine.

**Figure 8:**
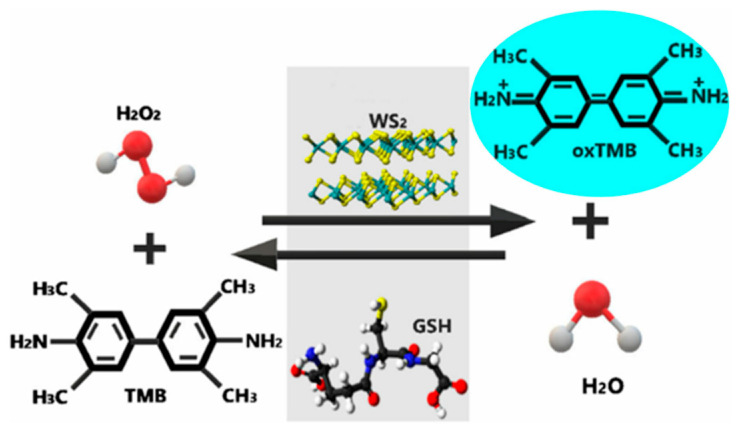
Colorimetric determination of GSH by tungsten disulfide. Reprinted with permission from Ref. [78], Copyright 2019, Royal Society of Chemistry.

Li et al. developed a colorimetric sensing platform based on WS_2_ based on the inhibitory effect of GSH on the nanozymatic catalytic activity of WS_2_ [78]. Figure 8 shows the sensing mechanism of the fabricated sensing platform, in which WS_2_ has the ability to oxidize TMB to turn the solution to blue. When GSH is present in the solution, it reacts with the TMB oxidation product to reduce the color of the solution, showing a change from blue to light blue and to colorless, thus achieving the purpose of colorimetric detection of GSH.

Previously, 2D TMO materials have exhibited great potential in energy storage and the preparation of efficient electrodes due to their abundant active sites and very short ion transport distance [79]. Recently, TMO materials have also attracted more and more attention due to their excellent electrical properties, optical properties, chemical and thermal stability, large specific surface area, and strong oxidation ability [80]. Li et al. developed an intracellular signal amplification method based on biodegradable MnO_2_ to monitor miRNAs with decreased gene expression in living cells. As MnO_2_ nanosheets are biodegradable, the toxicity to target cells is greatly reduced. After entering the cells, the degraded target miRNA-21 will trigger the degradation of MnO_2_ nanosheets and produce significant signal changes, which can be used to detect trace miRNA-21 in living cells [81]. In another case, Kim and co-workers prepared functional colorimetric hydrogen sensing materials, such as PdO/TiO_2_, PdO/MgO, PdO/TiO_2_, and PdO/SiO_2_, by an acid–base reaction between H_2_PdCl_4_ solution and substrate materials ZnO, MgO, TiO_2_, and SiO_2_ [82]. It was found that the PdO/ZnO hybrids have the best performance for the colorimetric sensing of hydrogen and can realize the rapid detection of hydrogen in daily life and industry.

### 3.3. MXene

Mxene material is a new 2D layered material, which has the characteristics of high conductivity, hydrophilicity, biocompatibility, large surface area, and easy functionalization, among which good biocompatibility and easy functionalization make it an excellent substrate for the preparation of functional materials for various applications [83]. Through the modification of Mxene materials with amino acids, proteins, and nucleic acids, a functional Mxene colorimetric sensor is designed for the sensitive and efficient detection of specific viruses, bacteria or biomolecules, which has a very wide application prospect.

Li et al. found that titanium carbide (Ti_3_C_2_) nanosheets show peroxidase-like activity, and the surface modification of Ti_3_C_2_ by ssDNA can enhance the catalytic activity of Ti_3_C_2_. At the same time, ssDNA also makes Ti_3_C_2_ a target recognition substance with better specificity and higher sensitivity and selectivity in the detection process [84]. Wang et al. proposed the label-free nanoplasmatic colorimetric detection of Ag^+^ based on polyacrylic acid (PAA)-modified Ti_3_C_2_ [85]. The PAA–Ti_3_C_2_ exhibited better stability in aqueous solution. When there are Ag^+^ ions in the solution, the PAA–Ti_3_C_2_ can effectively adsorb and reduce Ag^+^. When adjusting the concentration of Ag^+^, the solution showed different shades of brown, thus realizing the naked-eye visual colorimetric detection of Ag^+^ in the solution. The fabricated colorimetric Ag^+^ sensor exhibited a detection of 0.615 µM, which could be used for the rapid determination of Ag^+^ in drinking water.

**Figure 9:**
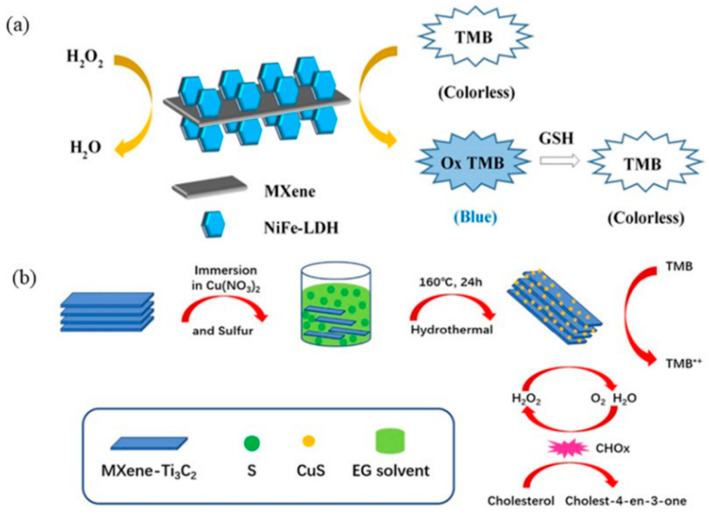
MXene-based colorimetric biosensors: (**a**) MXene@NiFeLDH visual colorimetric detection of GSH. Reprinted with permission from Ref. [86], Copyright 2020, American Chemical Society. (**b**) Ti_3_C_2_/CuS for colorimetric detection of cholesterol. Reprinted with permission from Ref. [89], Copyright 2019, Elsevier.

MXene-supported NiFe-layered double hydroxide (NiFeLDH) nanoparticles have been synthesized by an in situ coprecipitation method, and the created MXene@NiFeLDH has been used as a novel nanozyme for colorimetric sensing [86]. There are abundant hydroxyl groups on the surface of MXene materials. As a strong electron donor group, hydroxyl groups make the surface of Ti_3_C_2_ have a lot of negative charge, which promotes the binding of Ti_3_C_2_ with metal ions through electrostatic interaction and contributes to the nucleation of LDH. The synthesized MXene@NiFeLDH for the colorimetric detection of GSH is presented in Figure 9a, in which MXene@NiFeLDH catalyzes the oxidation of TMB to make the solution appear blue. When GSH was added, the good conductivity of Ti_3_C_2_ promoted the electron transfer of GSH and induced a solution color change from blue to colorless, thus realizing the visual colorimetric detection of GSH. In another study, Jin et al. loaded TiO_2_ quantum dots on the surface and edge of Ti_3_C_2_ MXene material through hydrothermal synthesis [87]. The formed TiO_2_–Ti_3_C_2_ revealed peroxidase-like activity for the catalytic oxidation of TMB. If GSH is added to the solution, it will reduce the oxidized state of TMB to fade the color of the solution to achieve the purpose of detection.

Li and co-workers demonstrated the synthesis of CuS–Ti_3_C_2_ hybrids by the hydrothermal synthesis of CuS NPs on Ti_3_C_2_ nanosheets [88]. The formed CuS–Ti_3_C_2_ hybrids had improved peroxidase-like activity compared to both CuS NPs and Ti_3_C_2_ nanosheets, revealing a potential application for the fabrication of colorimetric biosensors of cholesterol (Figure 9b). CuS–Ti_3_C_2_ can catalyze the oxidation of TMB by H_2_O_2_, and the solution color is different and the absorbance is different under different H_2_O_2_ concentrations. The colorimetric determination of H_2_O_2_ from the oxidation products of cholesterol can indirectly achieve the detection purpose according to the color of the solution.

On the basis of etching and exfoliation, Wang and colleagues synthesized blue-emitting uric acid (UA)-modified Ti_3_C_2_ composite quantum dots (UA@Ti_3_C_2_) by microwave treatment [89]. They found that the fluorescence of UA@Ti_3_C_2_ was quenched after adding 6-trinitrophenol (TNP). Based on this characteristic of UA@Ti_3_C_2_, a fluorescent probe of UA@Ti3C2 quantum dots was designed for the sensitive and selective colorimetric detection of TNP. In another case, Liu et al. prepared a fluorescence probe for the naked-eye detection of UA by using the fluorescence of GSH-modified Ti_3_C_2_ composite quantum dots (GSH@Ti3C2). As UA can quench the fluorescence of GSH@Ti_3_C_2_, thus the colorimetric detection of UA could be achieved via naked-eye observation [90].

### 3.4. MOFs

An MOF is formed by strong coordination interactions between metal ions/clusters and organic ligands. Due to its adjustable micropore structure, large specific surface area, and exposed active sites, it is highly useful for the preparation of artificial nanozymes. MOF-supported non-noble metal materials have become the most promising products to replace natural enzymes in biocatalytic reactions due to their low cost and good catalytic performance, which can replace noble metal nanomaterials in many catalytic reactions.

**Figure 10:**
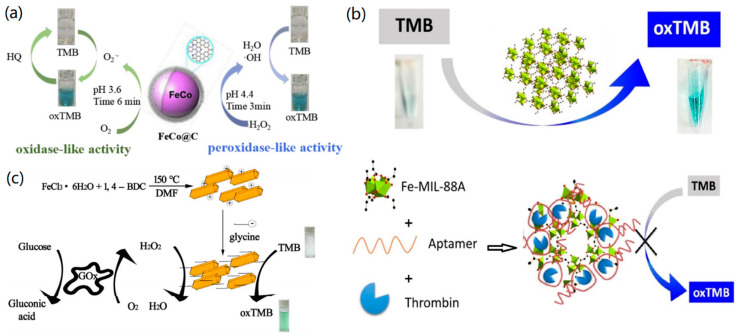
(**a**) FeCo@C catalyzed oxidation of TMB. Reprinted with permission from Ref. [91], Copyright 2019, Elsevier. (**b**) Aptamer-modified MOF for unlabeled thrombin. Reproduced with permission from Ref. [94]. Copyright 2016, Elsevier. (**c**) Fe-MIL-88A for thrombin. Reprinted with permission from Ref. [95], Copyright 2017, Elsevier.

For example, Wu and colleagues reported the synthesis of carbonized FeCo-ZIF (FeCo@C), which acted as a novel artificial nanozyme for the colorimetric detection of hydroquinone [91], as shown by the sensing mechanism in Figure 10a. The created FeCo@C had good activity and stability for TMB catalysis under acidic conditions. The fabricated colorimetric hydroquinone sensor exhibited a detection limit of 0.8 μM and a linear detection range of 1–30 μM. In another case, Yang et al. synthesized CoFe-decorated MOF material and developed a simple colorimetric detection method using TMB as a substrate [92]. Very recently, Wang and colleagues reported that two kinds of iron-based MOFs can be used for the colorimetric detection of H_2_O_2_ based on TMB [93]. Due to their different porous structures, the two iron-based MOFs had different catalytic properties, and if ascorbic acid (AA) was added to the TMB chromogenic process, the oxidation of TMB was inhibited to a certain extent. Therefore, the colorimetric detection of AA was achieved successfully.

However, MOF-based colorimetric platforms without specific recognition ability can only be used to detect H_2_O_2_, glucose, or some normal analytes, which limits the application of artificial nanozymes. To overcome this problem, a nucleic acid aptamer could be utilized, which has become an excellent recognition element because of its high stability, low cost, and easy functionalization. For instance, Wang et al. designed an aptamer-modified peroxidase-like catalytic activity sensing platform based on pure MOFs, and constructed a label-free colorimetric sensor for thrombin detection [94]. As shown in Figure 10b, Fe-MIL-88A can oxidize TMB to make TMB blue. When the mixture of thrombin aptamer and thrombin is added to the solution, the thrombin aptamer can selectively capture thrombin and form a stable intramolecular G-quadruplex structure (TA@ thrombin), which wraps on the surface of Fe-MIL-88A, thus blocking the electron transfer between Fe-MIL-88A and TMB and resulting in the color change from blue to colorless.

Dong and colleagues synthesized glycine-functionalized MOFs (glycine-MIL-53 (Fe)) by electrostatic interaction, and found that the formed glycine-MIL-53 (Fe) showed better catalytic activity and stability than pure MOF under alkaline or acidic conditions [95]. As shown in Figure 10c, the synthesized MIL-53 (Fe) is positively charged on the surface and can combine with negatively charged glycine in the aqueous phase to form glycine-MIL-53 (Fe), which can act as nanozymes for achieving the colorimetric detection of glucose. Yin and co-workers synthesized a protein-functionalized MOF by embedding bovine hemoglobin (BHb) into ZIF-8. The protein–MOF hybrid exhibited good stability and catalytic activity, and could be utilized to colorimetrically detect phenol in the presence of H_2_O_2_ [96]. According to Luo and co-workers, mixed valence Ce–MOF (Ce–BPyDC) had the activity of oxidase and peroxidase, which can be applied to the rapid and sensitive detection of AA via naked-eye observation [97].

MOF materials have a wide range of research prospects and application potential due to their microporous structure, high specific surface area and excellent catalytic performance. Polyoxometalate (POM) is an excellent oxidized metal cluster because of its nanometer scale, controllable composition, oxygen-rich surface with strong coordination ability and potential catalytic ability. The oxygen-rich surface of POM makes it a reliable precursor of MOF materials. Zhou and colleagues reported that columnar POM-supported MOFs can catalyze the oxidation of TMB by one-step self-assembly of benzotriazole and Cu^2+^ [98]. In addition, the enzyme–antibody conjugates can be prepared by functionalizing MOF materials with antibodies for colorimetric immunoassay. For instance, Wang et al. synthesized bifunctional MOF/antibody composites, called antimouse immunoglobulin G antibody (RIgG)@Cu–MOF, by integrating antibody RIgG and Cu–MOF [99]. The RIgG@Cu–MOF revealed high peroxidase-like activity and could be used for the fabrication of a colorimetric immunoassay of antigens. The functionalized MOF material synthesized by this method did not decrease or weaken the ability of RigG to capture antigens, but can protect RigG antibody from long-term storage and a high temperature environment. More importantly, the fabricated RIgG@Cu–MOF formed a sandwich structure with the captured antigen, which amplified the sensing signals and made the detection easier.

### 3.5. Two-Dimensional Metal Nanoplates

Nanoscale metal materials have a large specific surface area, excellent catalytic performance and good optical properties. In particular, nano-metal materials have great application prospects in colorimetric detection because of their excellent optical properties. It has been shown that functionalized 2D metal nanoplates can be used for colorimetric detection. Kiatkumjorn and colleagues modified Ag nanoplates with GSH and L-cysteine, and synthesized functionalized Ag nanoplates with high specificity to nickel ions (Ni^2+^). The combination of nickel ions and modifiers caused the aggregation of Ag nanoplates, resulting in a color change in the solution to achieve the purpose of detection [100].

**Figure 11:**
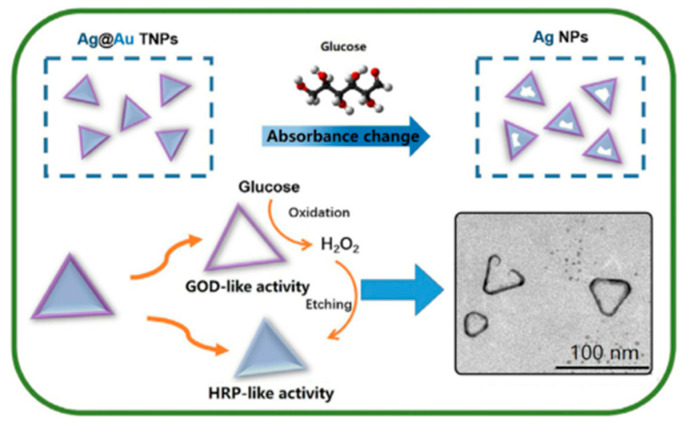
Schematic illustration of the colorimetric detection of glucose based on Ag@Au core/shell TNPs. Reprinted with permission from Ref. [102], Copyright 2019, Elsevier.

In addition, the unmodified 2D metal nanoplates also have good colorimetric properties. Bera et al. found that triangular Ag nanoplates have good selectivity to H_2_O_2_, the absorbance of Ag nanoplates will change under the etching of H_2_O_2_, and the color will also change visibly to the naked eye [101]. Based on this principle, Liu et al. developed a bimetallic Ag@Au hybrid material, a triangular nanoplate (Ag@Au core/shell TNPs) with Ag as the core and Au as the shell, which exhibited high specificity for glucose detection. As shown in Figure 11, the Au shell has peroxidase-like activity and can oxidize glucose, which mediates the etching of the silver core by H_2_O_2_, resulting in a change in absorbance, and the color becomes colorless instead of blue [102]. Similarly, Chang and colleagues achieved the colorimetric detection of Cu^2+^ through iodine-mediated Au triangular nanoplates. Through this basis, they achieved the colorimetric detection of chloramphenicol by modifying Au triangular nanoplates with nucleic acid aptamers [103].

## 4. Conclusions and Outlooks

In conclusion, we summarized recent advances in the design, fabrication, and applications of 2D material-based colorimetric biosensors. From the above case studies, it can be found that the sensing mechanisms of 2D material-based colorimetric biosensors include mainly the nanozyme-mediated TMB/H_2_O_2_ reaction, SPR change, and fluorescent switch. Some 2D materials, such as graphene, TMD, TMO, MXene, and metal nanoplates, have enzymatic mimic properties, and therefore could be utilized for the fabrication of TMB/H_2_O_2_ reaction-based colorimetric sensors and biosensors. In addition, the conjugation of other metal NPs such as Pt, Pd, Fe, Co, Cu, and others could promote the catalytic activity of 2D materials, resulting in improved sensitivity and selectivity of the fabricated colorimetric biosensors. Based on the SPR change of AuNPs and AgNPs, 2D materials can bind with these color NPs to form hybrid nanomaterials. After the binding of analytes onto the formed colorimetric sensor platforms, the change in SPR induces the change in the solution color for achieving colorimetric detection. Besides, some fluorescent dyes and Schiff bases could be utilized for the modification of 2D materials in order to fabricate colorimetric biosensors of small molecules and anions. Therefore, by tailoring the structure, surface modification, and hybridization with other nanomaterials, 2D materials could be excellent candidates for the fabrication of high-performance colorimetric sensors and biosensors.

It is also very important to understand the sensing mechanism of various 2D material-based colorimetric biosensors. Based on the above introduction and discussion, it can be found that 2D material-based colorimetric sensing mechanisms include mainly SPR change, enzyme and mimic enzyme catalysis, and fluorescence switch. Through the conjugation of noble metal nanomaterials with 2D materials, it is possible to fabricate colorimetric biosensors by using the SPR change mechanism. The fabricated colorimetric biosensors could be utilized to determine small molecules, DNA, proteins, and others. However, most of the 2D material-based colorimetric biosensors are based on the catalysis of nanozymes. Two-dimensional nanomaterials are excellent nanozymes for the construction of colorimetric biosensors, and the addition of other nanozymes into 2D materials enhanced the catalytic activity of hybrid materials, decreased the detection limit, and improved the sensitivity and selectivity of biosensors due to the synergistic effects.

Two-dimensional materials have been widely utilized for the fabrication of colorimetric sensors and biosensors; however, a few drawbacks of 2D materials should be considered when constructing colorimetric sensors and biosensors. For instance, 2D materials under different detection environments (acidic, basic, and biological systems) are relatively unstable and easy to aggregate, affecting the sensing performance of sensors. The reproducibility of fabricated sensors and biosensors is not as good as other electrochemical, fluorescent, and electronic sensors in some cases. In addition, 2D materials are not as economic as other traditional nanomaterials (such as metal and metal oxide nanoparticles) for the fabrication of colorimetric sensors and biosensors, which hinders their practical applications.

Although great improvements have been made in this promising research field, there are still some challenges and problems that should be faced. First, compared to traditional AuNPs and AgNPs, most 2D materials are expensive and hard to functionalize, which hinders the practical applications of fabricated 2D colorimetric biosensors. It is necessary to develop facile strategies to mediate the synthesis and functionalization of 2D materials to overcome this problem. Second, the dispersity of 2D materials is another problem for the fabrication of colorimetric biosensors. However, this problem could be solved by conjugation with water-soluble biomolecules. This kind of effort will make it possible to achieve the biomodification of 2D materials with biomolecules and antibodies/antigens, promoting their biomedical applications, for instance, the detection of bacteria and viruses, in water, air, and blood systems. Third, it has been reported that single-atom nanozymes exhibited a great improvement on catalytic activity materials, and therefore it is potentially interesting to synthesize single-atom (Pt, Pd, Fe, Co, and others) nanozymes on the surface of 2D materials to create hybrid 2D nanozymes for colorimetric biosensors. The effects of the loading and the density of single atoms of layered 2D surfaces on the sensor performance could be studied by control experiments. Fourth, 2D materials could be utilized for the fabrication of colorimetric fluorescent biosensors for disease diagnostics and cancer therapy, especially for real-time in vivo and in vitro analysis of disease factors, cancer cells, and biomarkers. Fifth, 2D material-based colorimetric sensors and biosensors are highly required for society and country safety, for instance, through the ultra-sensitive monitoring of toxic gases and chemicals, restricted drugs, and dangerous explosives/weapons. We believe 2D materials will contribute more to the fields of colorimetric sensors and biosensors with the quick development of nanotechnology, materials science, and biological science in the future.

## Figures and Tables

**Table 1:** The sensing mechanism and detection substances of various material-based colorimetric sensors.

Materials	Interaction	Detection Object	Ref.
Ag NPs	SPR change	Proteins	[32]
Au NPs	SPR change	Metal ions	[33]
Ag NPs	SPR change	Cu^2+^	[34]
2D metal oxides	Enzyme and nanozyme catalysis	Glucose	[38]
MoO_3_ NPS	Enzyme and nanozyme catalysis	ACP	[39]
Schiff base derivatives	Fluorescent switch	Al^3+^	[43]
DNA templates	Fluorescent switch	Au/Ag Nanoclusters	[44]
Bifunctional probes	Fluorescent switch	F^−^	[45]
Chelated Schiff base	Ligand–receptor binding	Fe^2+^ and Fe^3+^	[46]
Multifunctional Schiff base	Ligand–receptor binding	Cu^2+^	[47]
Multifunctional Schiff base	Ligand–receptor binding	CN^−^	[48]
PHCS	Photonic crystals	Bacterial contaminants	[53]
1D PC	Photonic crystals	Humidity	[54]

## Data Availability

Not applicable.
